# Evolution of Pore Structure and Damage Mechanism Analysis of Cement–Silt-Modified Eolian Sand Under Freeze–Thaw Cycles

**DOI:** 10.3390/ma18163800

**Published:** 2025-08-13

**Authors:** Xunchang Li, Chenyu Miao, Zhengzheng Shi

**Affiliations:** School of Geological Engineering and Geomatics, Chang’an University, Xi’an 710054, China; 2023126126@chd.edu.cn (C.M.); 2022126129@chd.edu.cn (Z.S.)

**Keywords:** modified eolian sand, freeze–thaw cycle, pore structure, microstructure

## Abstract

This paper explores how freeze–thaw cycles affect the mechanical properties and pore structure in cement–silt-modified eolian sand. The study addresses freeze–thaw durability issues for cold, arid region engineering. We tested samples with 5% and 8% cement content at a 3:7 silt-to-sand ratio using freeze–thaw cycling, unconfined compression tests, and an SEM. Gray relational analysis quantified pore–strength correlations. The results indicated that after 10 freeze–thaw cycles, the strength of the 5% cement content samples decreased by over 80%, while the strength of the 8% cement content samples decreased by approximately 25%. The total number of pores increased with the number of freeze–thaw cycles. The proportion of large pores also continued to rise. The pore shapes degraded from circular/elliptical to elongated. The pore orientation shifted from a concentrated distribution (90°~105°) to a more random dispersion. The proportions of large pores (with correlation coefficients exceeding 0.80) and extremely low abundance pores (with correlation coefficients exceeding 0.82) served as the primary microstructural parameters affecting strength loss. This research uncovered the freeze–thaw damage mechanism of cement–silt-modified eolian sand. It provides a theoretical foundation for material design in cold and arid region roadbed engineering and for enhancing the freeze–thaw resistance of modified materials.

## 1. Introduction

Eolian sand is a mixture of sand and gravel formed by wind action. Due to its unique physical and chemical properties, it has become an important construction material in desert regions. However, due to its irregular particle shape, low strength, and almost zero cohesion, its application in engineering faces certain challenges. Despite this, with the continuous deepening of research on eolian sand, researchers have proposed various improvement methods, gradually overcoming these limitations and making the engineering application prospects of eolian sand increasingly broad. Research on eolian sand began early abroad, primarily focusing on road alignment and sand prevention and control, such as the design of sand barriers and planting vegetation to protect highways in the Middle East. With further research, eolian sand has gradually been applied to roadbed filling projects. Amhadi [1] improved eolian sand by blending cement, fly ash, and artificial sand and gravel, thereby optimizing its strength, compaction, and load-bearing capacity; Ghrieb [2] improved the particle size distribution of eolian sand by adding artificial mechanically produced sand and evaluated the road performance of cement-modified eolian sand; Alkarni [3] studied the improvement in the strength and shear strength of dune sand mixed with cement. In recent years, China has achieved a series of practical results in the application research of eolian sand [4,5,6], particularly in highway and railway construction. For instance, the Taklamakan Desert Highway and the Chifeng-Tongliao Expressway in Inner Mongolia have both utilized eolian sand as roadbed fill material; the Hezuo Railway employs eolian sand for roadbed construction, combined with vegetation-based sand barriers to prevent wind erosion damage. These studies have provided important practical experience and theoretical support for the engineering application of eolian sand in desert regions.

Research on the improvement in eolian sand mainly focuses on physical and chemical modifications. For physical modification, incorporating different types of fine- or coarse-grained soils optimizes the particle size distribution of eolian sand. This improvement enhances its mechanical properties. Studies show that adding silt helps increase the maximum dry density and cohesion. It also significantly optimizes particle gradation [7,8,9,10]. At different ratios, the silt content significantly impacts the shear strength and compaction characteristics. An appropriate silt content enhances economic benefits [11]. In terms of chemical modification, adding inorganic binders such as cement and fly ash enhances the mechanical properties of eolian sand. This occurs through hydration reactions. These reactions produce gels and crystals [12,13]. The cement content and curing conditions significantly affect the unconfined compressive strength of eolian sand. Increasing the cement content improves strength [14]. However, strength varies significantly under different curing conditions [15,16]. Studies also show that the mechanical properties of cement-modified eolian sand deteriorate during freeze–thaw cycles [17].

Recent engineering construction advances in cold and arid regions have increased research interest in the freeze–thaw durability of modified eolian sand. Studies show that cement-modified eolian sand develops microcracks in its hydration products under temperature gradients. Freeze–thaw forces also restructure pores. This significantly weakens the material’s structural stability [18,19]. Zhang [18] systematically studied how freeze–thaw cycles affect the shear strength and pore structure of salt-tolerant cement-modified eolian sand. He revealed the strength degradation mechanism under the combined effects of salt swelling and freeze swelling. Ruan [20] investigated the enhancing effect of the basalt fiber content on the splitting tensile strength of cement-modified eolian sand after freeze–thaw cycles. Hua [21] combined laboratory experiments with numerical simulations to analyze the inhibitory effects of the cement content and curing age on freeze–thaw settlement. He also verified the freeze–thaw resistance mechanism of the cement-soil matrix. Li, J [22] discovered that the crack width and length of cement-modified eolian sand change with an increasing number of freeze–thaw cycles. Yi, F [23] developed a constitutive model for freeze–thaw damage in modified eolian sand, which has fractal characteristics.

Through engineering practices in permafrost regions, researchers have found that stabilizing soil with cement alone cannot satisfy long-term durability requirements under repeated freeze–thaw cycles. Therefore, composite modification methods have been developed to enhance the material’s freeze–thaw resistance [24,25,26,27]. Jianwen B [28] found that adding eolian sand to concrete can effectively suppress and delay freeze–thaw damage. Chen Y [29] used eolian sand and fly ash to stabilize saline soil. After seven freeze–thaw cycles, the material’s strength increased 234 times compared to that of untreated soil. Assel J [30] evaluated the impact of freeze–thaw cycles on the strength and durability of calcium sulfoaluminate (CSA) cement-modified sand. Baldovino, J. [30] pointed out that freeze–thaw cycles might weaken the effect of cement hydration products in filling soil pores and particles, leading to microcracks. Related studies have shown that adding silt can optimize gradation and improve the initial strength of the material [31,32,33]. The cement-hydration-formed gel-like skeleton and the silt that fills pores act together. They may exhibit nonlinear degradation behavior under freeze–thaw stress [34]. However, the current research on cement–silt-modified eolian sand is relatively limited. There is especially a lack of studies on the mechanical properties of this material under freeze–thaw cycles.

This study aims to uncover how freeze–thaw cycles affect the compressive strength and pore structure of cement-modified eolian sand through systematic experimental analysis. It uniquely focuses on pore morphology and uses cement–silt composites. We used unconfined compressive strength tests and SEM analysis. We thoroughly analyzed the test results and multi-dimensional pore characteristics. This explored the complex evolution of pore structure under freeze–thaw cycles and its effect on freeze–thaw resistance. This research holds significant theoretical and practical value. It advances resource utilization in desert areas, ensures the safety of major engineering projects, and promotes regional sustainable development.

## 2. Materials and Methods

### 2.1. Test Material

**Figure 1:**
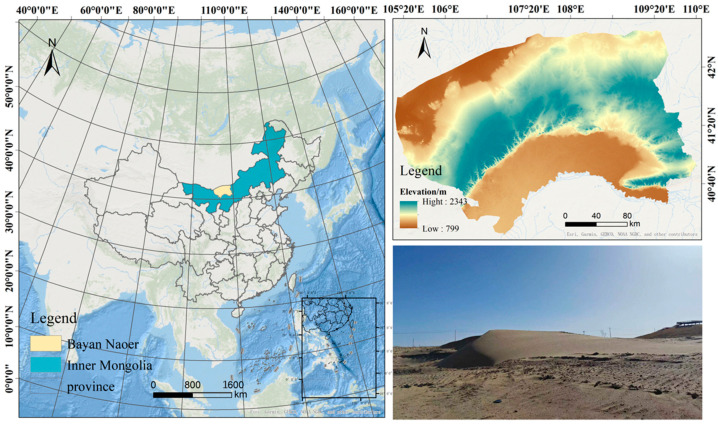
Overview map of the research area.

**Figure 2:**
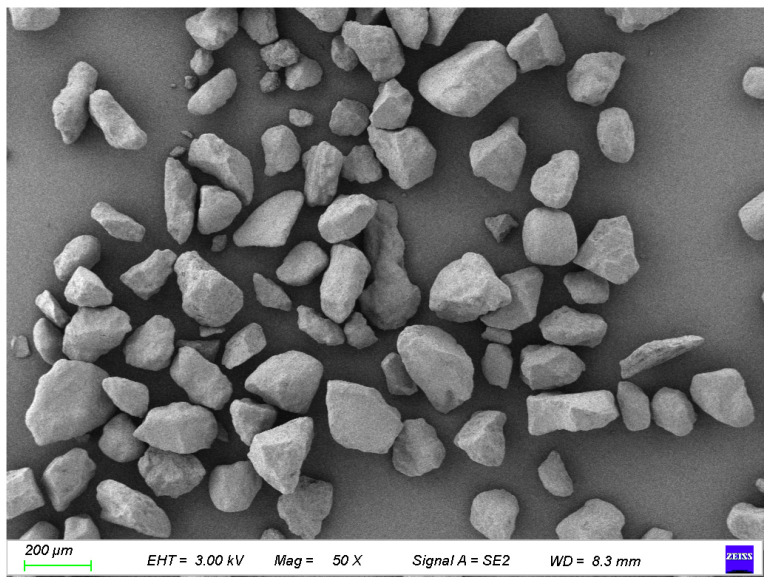
SEM image of eolian sand.

The eolian sand used in this experiment was sourced adjacent to the Baotou–Huinong section of the Baotou–Yinchuan High-Speed Railway. The sampling site is located in a piedmont alluvial–pluvial plain geomorphic unit. After sieving, the fundamental physical properties of the eolian sand and the silt were determined according to the Geotechnical Testing Procedures for Railway Engineering (TB 10102-2023) [35]. Table 1 presents the results. Figure 1 shows the overview map of the study area. Figure 2 presents the SEM image of the pure eolian sand. The cement used was Baoji Conch P·O 42.5 Portland cement. Its chemical composition is listed in Table 2.

First, according to the ‘Standard for Soil Test Methods’ (GB/T 50123-2019) [36], eolian sand and silt were sorted using a 2 mm standard sieve. After sieving, the soil samples were placed in a constant temperature oven at 105 °C for 24 h to remove moisture. The dried eolian sand, silt, and cement were then accurately weighed. The mixed materials were dry-mixed, then water was added, and the mixture was thoroughly stirred to ensure the cement was evenly distributed in the test samples. After forming the samples, standard curing was conducted to ensure the specimens achieved the required mechanical properties for testing.

**Figure 3:**
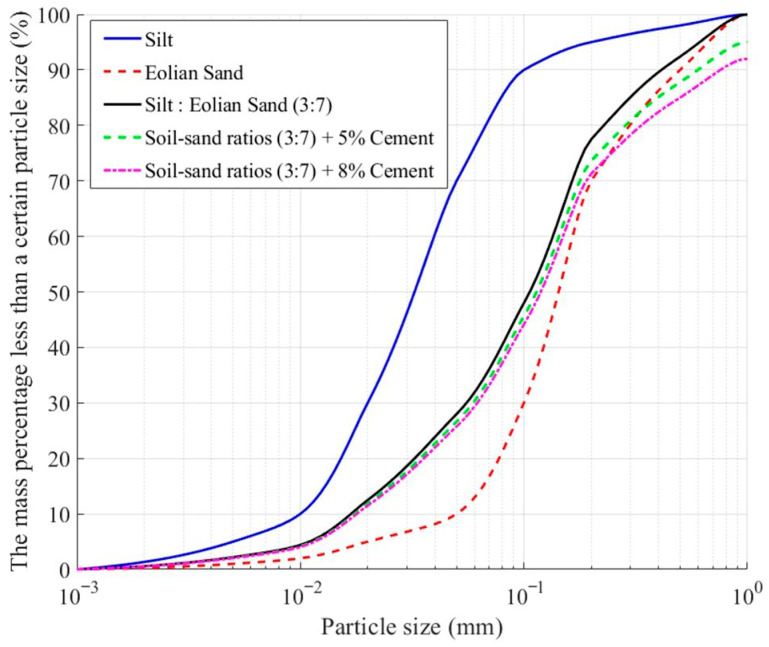
Particle size distribution curves of eolian sand, silt, and modified eolian sand.

By reasonably setting the proportions of cement, silt, and eolian sand, the particle gradation of the material can be optimized to enhance its freeze–thaw stability. At the same time, under the premise of ensuring material performance, the amount of cement is minimized to reduce costs. The mix design scheme is shown in Table 3. The particle size distribution curves for pure eolian sand, pure silt, and modified eolian sand (soil–sand ratio 3:7, cement content 5% and 8%) are shown in Figure 3.

### 2.2. Sample Preparation

**Figure 4:**
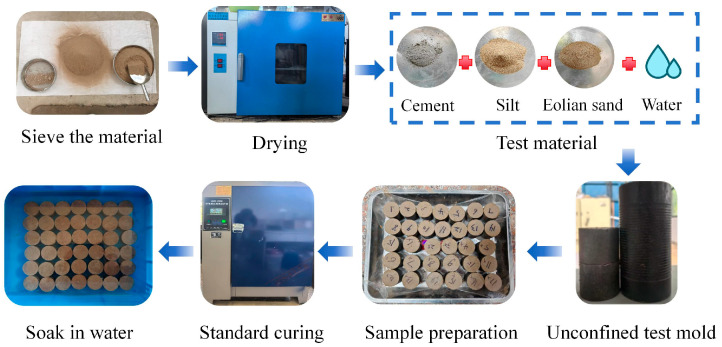
Sample preparation process.

Following the Standard for Soil Test Methods (GB/T 50123-2019) [36], a layer of Vaseline was applied to the inner wall of the Φ50 mm × H50 mm unconfined test mold. The mold was then filled with modified eolian sand. Static compaction to 95% density was achieved using an unconfined forming press-de-molding machine, with pressure maintained for 1 min. After mold removal, the specimens were cured for 24 h prior to de-molding. The de-molded samples were wrapped in plastic wrap and transferred to a constant temperature–humidity chamber for standard curing at 22 °C and ≥95% relative humidity (7 days). After curing, the samples were immersed in 22 °C distilled water for 24 h for saturation. The water level was maintained at approximately 2.5 cm above the sample tops to ensure full saturation. Following soaking, the excess water was wiped from the sample surfaces, followed by weighing. For this test, three parallel samples were prepared per test condition. With 17 distinct test conditions, the total reached 51 samples. Figure 4 illustrates the laboratory sample preparation process.

### 2.3. Test Methods

The test methods used in this study include freeze–thaw cycle tests, unconfined compressive strength tests, and scanning electron microscope (SEM) tests. Each method was conducted strictly according to the relevant standards.

#### 2.3.1. Freeze–Thaw Cycle Test

**Figure 5:**
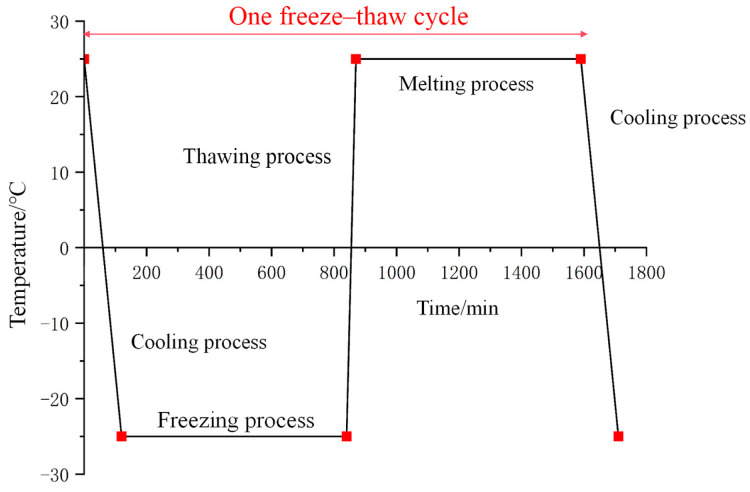
The freeze–thaw cycle temperature–time curves of modified eolian sand with different cement contents (5% and 8%).

Meteorological data from the Bayan Nur region shows that minimum temperatures from November to March typically remain above −25 °C, while maximum temperatures can reach approximately 25 °C. Following the freeze–thaw test method in the Highway Geotechnical Test Procedures (JTG 3430-2020) [37], we selected a temperature range of −25 °C to 25 °C for freeze–thaw cycle testing. After soaking, the samples undergo freeze–thaw cycles in a fully automatic low-temperature testing chamber (±0.5 °C temperature accuracy). Each cycle consists of 12 h at −25 °C (freezing) and 12 h at 25 °C (thawing). After completing the cycles, we test the compressive strength. Figure 5 shows the temperature–time curve.

#### 2.3.2. Unconfined Compressive Strength Test

This test was conducted according to the unconfined compressive strength test method outlined in the ‘Standard for Soil Test Methods’ (GB/T 50123-2019) [36]. The loading operation was performed using a WCY-1 stress–strain-controlled unconfined compression apparatus manufactured by Nanjing Zhongzhi Rock Control Technology Co., Ltd. The loading rate of this instrument is approximately 1 mm/min, with a maximum load capacity of 3 kN, a range of 50 mm, and an accuracy of 0.01 mm. The sample is placed on the lifting platform of the unconfined compression tester for the compressive test. The center of the sample must be aligned with the vertical loading point to avoid experimental errors caused by uneven loading. Throughout the test, high-resolution imaging equipment captures real-time surface deformation of the sample. Loading is stopped when the axial strain reaches 6% or when sample failure occurs, and the maximum axial stress at the point of failure is recorded.

#### 2.3.3. SEM Testing

**Figure 6:**
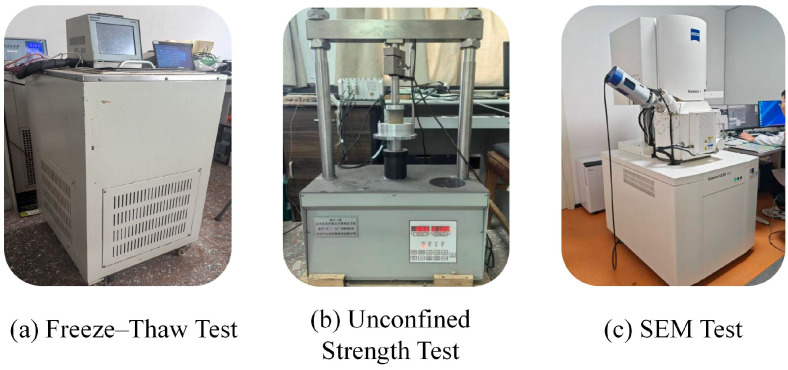
Tested instruments.

The scanning electron microscope (SEM) scans a sample’s surface with an electron beam. It collects signals from interactions between electrons and sample atoms. These signals include secondary electrons, backscattered electrons, and X-rays. The signals provide information about the sample’s surface morphology and elemental composition. An SEM achieves nanometer-scale resolution. It also provides detailed three-dimensional surface images and elemental analysis. This experiment used the Gemini SEM 360 field emission scanning electron microscope produced by Carl Zeiss GmbH in Germany. The experimental procedure followed the ‘General Rules of Analytical Methods for Scanning Electron Microscope’ (JY/T 0584-2020) [38]. Figure 6 shows the test instruments.

## 3. Test Results

### 3.1. Preliminary Analysis of Compressive Strength

**Figure 7:**
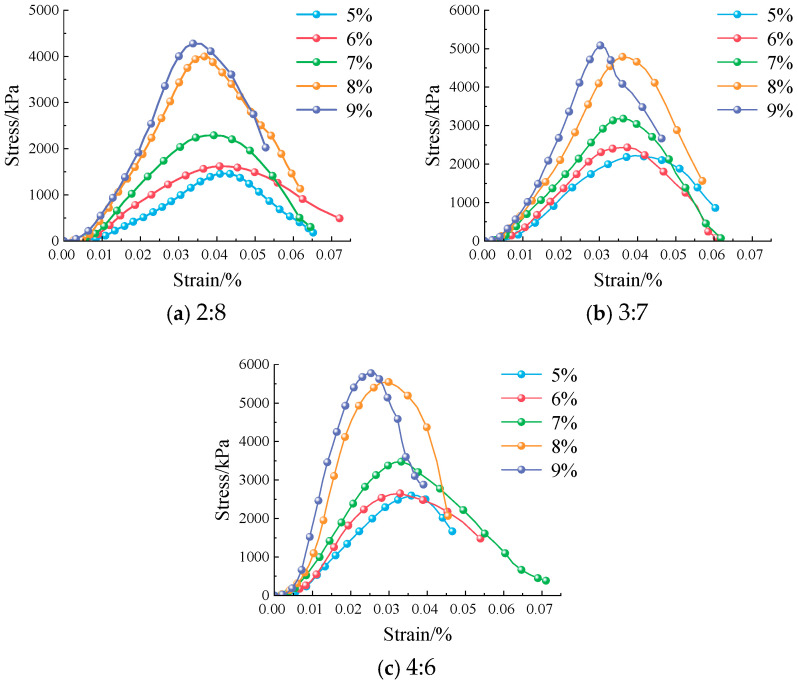
Stress–strain curves for different cement contents after 7 days.

Before conducting the freeze–thaw cycle test, a pre-test was performed to analyze the strength of the modified eolian sand. As shown in Figure 7, specimens with 8% cement content exhibit a gradual stress decline after peak stress. This decline occurs with increasing strain. In contrast, specimens with 9% cement content show a rapid stress drop after the peak. Their curve is sharp and transient. These observations indicate that the 9% cement material fails abruptly at maximum stress. It lacks the capacity for further deformation. Compared to the 9% cement specimens, those with 8% cement have better ductility. This ductility provides higher plastic deformation capacity and resistance to dynamic loads.

In addition, an excessively high silt proportion causes clay particle aggregation, while an excessively low silt proportion cannot meet strength requirements [31,33,39]. Both conditions reduce structural stability. Based on pre-test results and references [31,33,40,41,42], specimens with a silt-to-eolian-sand ratio of 3:7 and 8% cement content are selected for the freeze–thaw cycle study. The control group uses the same soil-to-sand ratio but 5% cement content. The freeze–thaw cycles are set to 1, 2, 4, 7, and 10. Table 4 shows the mix design scheme.

### 3.2. Effect of F-T Cycles on Compressive Strength of Modified Eolian Sand

**Figure 8:**
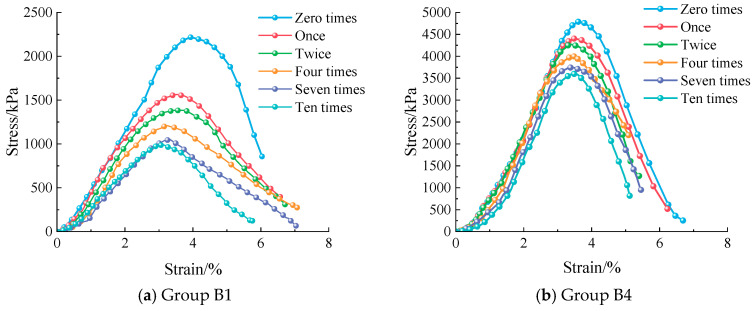
Stress–strain curve diagram for different F-T cycle numbers.

As the number of freeze–thaw cycles increases (Figure 8), both Group B1 and Group B4 show declining mechanical properties, but with significant differences. The peak stress and peak strain of both groups decrease continuously. Failure occurs earlier, and the ascending curve slope decreases and shifts left. In Group B1, the slope fluctuates sharply, indicating that low-cement materials are more sensitive to freeze–thaw cycles. In contrast, Group B4 shows smoother slope changes, suggesting that the higher cement content improves frost resistance effectively. A higher cement content intensifies the hydration reaction, producing more hydration products. These products densify the material’s internal pore structure [11,29], reducing water migration channels in both number and size. Consequently, during freeze–thaw cycles, water movement within the material is restricted, limiting frost heave damage. This process enhances the freeze–thaw resistance of the modified material. However, an excessively high cement content increases material brittleness during freeze–thaw cycles [43,44]. A high cement content densifies and rigidifies the cement matrix. This dense matrix lacks sufficient toughness to accommodate freeze–thaw-induced volumetric changes, causing brittle failure.

The compressive strength test quantified performance changes during freeze–thaw cycles (Figure 8). Group B1 had an initial strength of 2.2 MPa. After 10 freeze–thaw cycles, its compressive strength dropped sharply to less than 1 MPa, showing a strength loss exceeding 80%. In contrast, Group B4 had a higher cement content, forming a more stable structure. Its initial strength was 4.7 MPa. After 10 freeze–thaw cycles under identical conditions, the compressive strength remained at 3.6 MPa, with only a 25% reduction.

**Figure 9:**
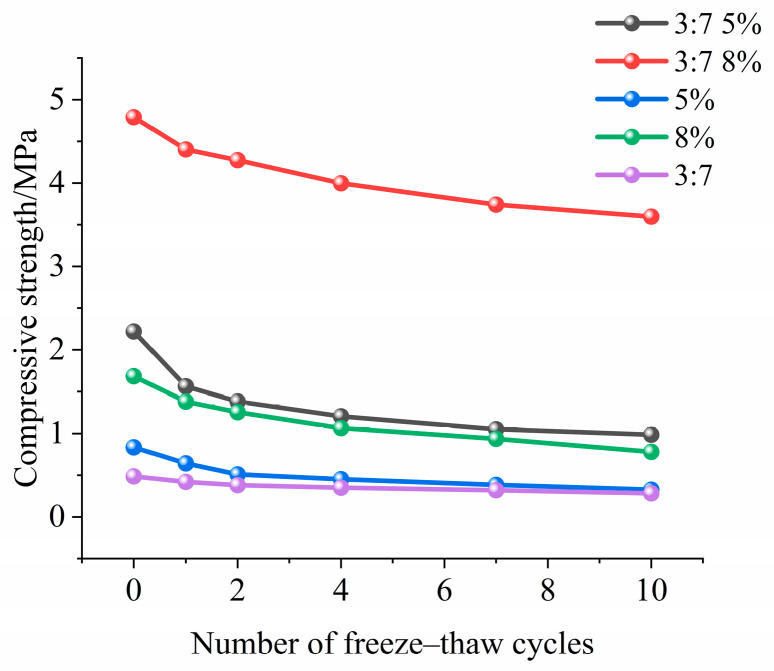
The effect of F-T cycles on unconfined compressive strength.

Compared to unmodified eolian sand with 8% cement, the composite material showed a 184% increase in initial compressive strength and a 281% improvement in freeze–thaw resistance (Figure 9). For sand with 5% cement alone, the initial compressive strength increased by 197%, and freeze–thaw resistance improved by 200%. Additionally, cement-modified eolian sand exhibited significantly higher compressive strength than silt-modified sand. These results indicate that a higher cement content enhances both the initial compressive strength and freeze–thaw resistance of the material.

### 3.3. Qualitative Analysis of Microstructure

**Figure 10:**
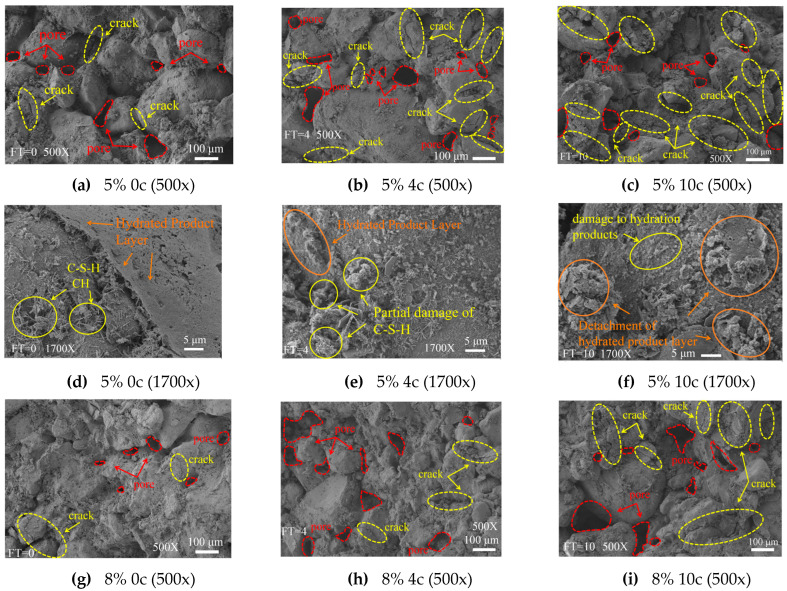
SEM image of modified eolian sand.

Figure 10 shows SEM images of modified eolian sand after 0, 4, and 10 freeze–thaw cycles. As the freeze–thaw cycles increased, the pores and cracks gradually expanded in samples with both the 5% and 8% cement content. In the 5% cement sample before freeze–thaw cycles, hydration products exhibited a dense structure. Ettringite needles and calcium silicate hydrate plates were uniformly distributed, forming a compact matrix. After four cycles, some hydration products degraded. Although ettringite needles and calcium silicate hydrate plates remained visible, initial pores and cracks appeared. After 10 cycles, further deterioration occurred. The number of ettringite needles and calcium silicate hydrate plates significantly decreased. The structure loosened, with prominent pores and cracks, leading to reduced compactness.

The SEM images revealed significantly fewer pores and cracks in samples with an 8% cement content than in those with a 5% cement content. This indicates that the 8% cement content significantly enhances the freeze–thaw resistance of modified eolian sand. In contrast, samples with the 5% cement content exhibited increased pores and cracks after multiple freeze–thaw cycles, indicating poorer freeze–thaw resistance. The lower cement content likely failed to fill voids between particles, making the material more vulnerable to freeze–thaw damage.

### 3.4. Quantitative Analysis of Microstructure

After electron microscope testing, we performed image processing and quantitative analysis using Image Pro Plus software (Version 6.0). The analysis focused on pore morphology, size, and distribution characteristics. For each sample, we imaged three different regions. Each group contained 10 consecutive slices to prevent local errors. All data analysis steps were strictly followed by standard operating procedures, ensuring result accuracy and reproducibility.

The image analysis process includes these steps.

**Figure 11:**
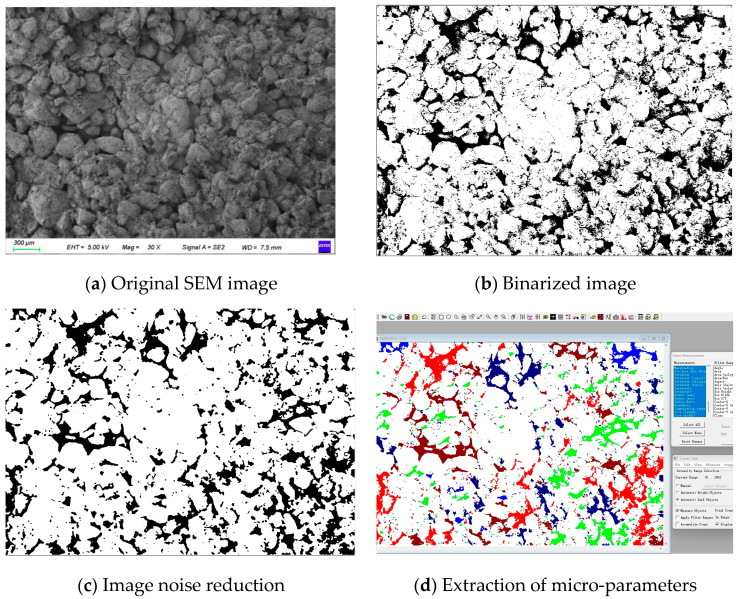
Image processing and parameter extraction process.

1. Image Preprocessing: Import measured SEM images (scale: 300 μm) into Image Pro Plus software. Adjust grayscale to enhance contrast (Figure 11a).

2. Threshold Setting and Segmentation: Perform initial segmentation via the global threshold method. Set the threshold range to 100–255 based on the grayscale histogram (Figure 11b).

3. Artifact Removal: Apply median filtering to reduce noise (Figure 11c).

4. Pore Measurement: Extract pore micro-parameters (area, length, number, orientation angle) (Figure 11d). Among them, different colors represent pores within different range intervals.

5. Visualization Analysis.

#### 3.4.1. Pore Distribution Changes

The average diameter is calculated from the diameter of a circle with an equivalent pore area. The calculation method is given in Equation (1):

(1) $$D=\frac{\sqrt{S}}{\pi }$$

In the formula, D represents the average pore diameter (μm), and S denotes the area of a circle equivalent to the pore area.

**Figure 12:**
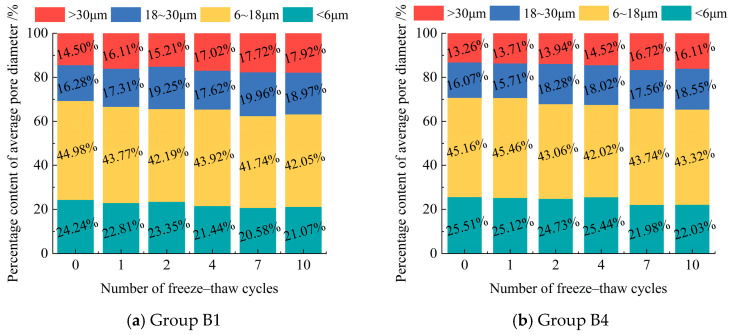
Percentage of pore diameter content under different F-T cycles.

Pore diameters are classified into four ranges based on their distribution: <6 μm, 6–18 μm, 18–30 μm, and >30 μm. The number and percentage of pores in each diameter range are calculated for different freeze–thaw cycles. The results are presented in Figure 12.

As the number of freeze–thaw cycles increased, the total pore count in the modified eolian sand grew continuously. The proportion of pores larger than 18 μm rose, while pores smaller than 18 μm decreased overall. During freeze–thaw cycles, the pore diameters in the modified eolian sand were mainly between 0 and 18 μm. Pores larger than 30 μm had the lowest proportion. These findings match results from studies [42,45]. These studies show that more freeze–thaw cycles increase large pore proportions but decrease small pore proportions. Frost heave pressure expands small pores into larger ones. It also damages the material’s fine structure. This disrupts the dense pore network and reduces small pores [46]. During ice melt, large pores may shrink and small pores may grow. But the overall large pore count still rises compared to that of the initial structure.

Comparing the pore distribution between the two groups with different cement contents shows that samples with 8% cement have a significantly lower proportion of pores larger than 18 μm than those with 5% cement. This indicates that increasing the cement content effectively suppresses the formation of larger pores [47,48]. This suppression enhances the soil’s compactness, which improves its freeze–thaw resistance and compressive strength. These results agree with the findings in Section 3.2.

#### 3.4.2. Evolution of Pore Morphology

Pore abundance is the ratio of the pore’s equivalent short axis to its long axis. This ratio characterizes the geometric shape of the pore and ranges from 0 to 1. When the abundance value approaches 1, the pore is nearly circular. When the value approaches 0, the pore has a needle-like or elongated shape.

(2) $$C=\frac{B}{L}$$

In the equation, C represents pore abundance, and B and L denote the short axis and long axis of the pores, respectively.

**Figure 13:**
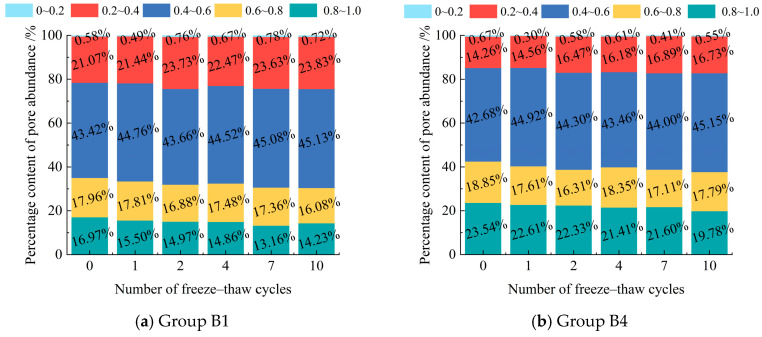
Percentage content of pore abundance under different F-T cycles.

As shown in Figure 13, freeze–thaw cycles reduce the abundance of both sample groups in the 0.6–1.0 range. This range indicates nearly circular pores. Conversely, abundance increases in the 0.2–0.6 range, which indicates elongated pores. These changes suggest that freeze–thaw cycles degrade pore shapes from circular/elliptical to elongated. This degradation primarily results from tensile stress generated during freeze–thaw processes. This stress gradually elongates or expands pore shapes. Circular or elliptical pores distribute stress more evenly under freeze–thaw conditions than elongated pores do. This reduces stress concentration at pore walls and lowers the risk of microcrack propagation [49,50,51].

When the cement content increases from 5% to 8%, pore abundance in the 0.8–1.0 range significantly rises—from 14.23 to 16.97% to 19.78–23.54%. Conversely, pore abundance in the 0.2–0.4 range decreases, dropping from 21.07–23.83% to 14.26–16.73%. These changes indicate that the higher cement content shifts pore shapes from elongated to circular or elliptical forms [48,51]. Cement hydration products play a key role by inhibiting large pore formation and maintaining pore roundness.

#### 3.4.3. Pore Orientation

Pore orientation describes the distribution of pore orientation angles from 0° to 360°. This distribution characterizes the direction of microcracks caused by microscopic damage. We use probability entropy to quantify the pore orientation for each failure mode. It is defined as follows:

(3) $$H=-\sum_{i=1}^{n}P_{i}{log}_{n}(P_{i})$$

In the formula, $P_{i}$ represents the percentage of the pore volume in a specific directional interval. For example, i = 1 corresponds to the 0–10° range.

**Figure 14:**
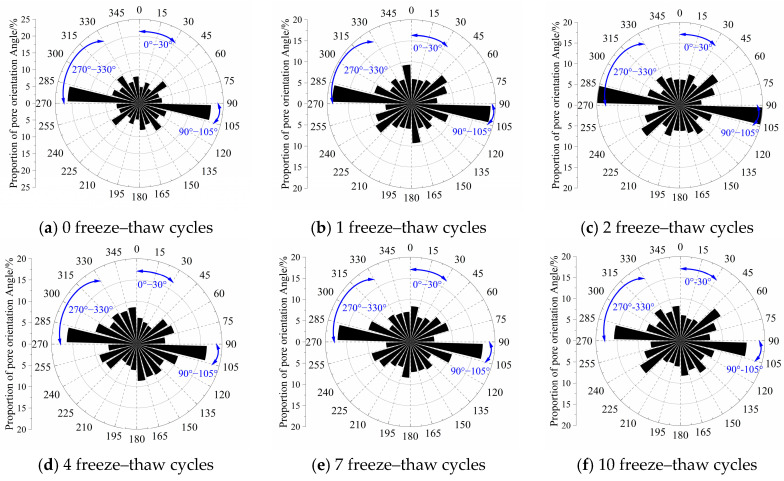
B1 Distribution of pore orientation angles according to different F-T cycles.

**Figure 15:**
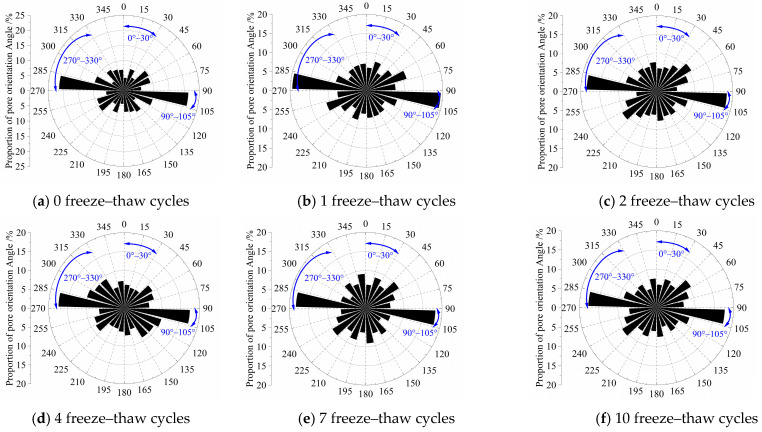
B4 Distribution of pore orientation angles according to different F-T cycles.

Modified eolian sand shows strong pore orientation characteristics before and after freeze–thaw cycles (Figure 14 and Figure 15). Particle orientation angles mainly concentrate in the 90–105° range, indicating directional alignment. This alignment relates to the gravity compaction direction during sample preparation. As freeze–thaw cycles increase, Group B1’s orientation probability entropy rises from 0.9606 to 0.9814, while Group B4’s increases from 0.9560 to 0.9765. Pore orientation angles gradually distribute more evenly across all ranges, with each angle range approaching balanced proportions. These changes demonstrate material evolution from a layered structure (oriented pores) to a loose structure (random pores).

Initially, pores concentrate in the 90–105° range (vertical direction). After freeze–thaw cycles, pore diffusion develops symmetric preference (Figure 11 and Figure 12). Proportions in low-angle (0–30°) and high-angle ranges (270–330°) increase with freeze–thaw cycles due to horizontal stress release. For low-angle ranges, the 5% cement group increases by 2.09%, 1.53%, 0.23%, 3.03%, and 2.59%, while the 8% cement group rises by 2.56%, 1.83%, 0.84%, 2.07%, and 2.31%. For high-angle ranges, the 5% cement group grows by 2.38%, 2.06%, 1.44%, 0.98%, and 1.64%, versus 3.36%, 1.54%, 2.13%, 0.59%, and 1.68% for the 8% cement group. After 10 freeze–thaw cycles, oblique-angle pores (45°, 135°, 225°, 315°) increase 6–12% against non-freeze–thaw samples, confirming particle shear sliding from ice expansion pressure.

### 3.5. Relationship Between Pore Parameters and Compressive Strength

Gray relational analysis (GRA) evaluates the correlation between system factors. Based on gray system theory, this method works well for small samples and incomplete information. GRA determines the correlation strength by calculating associations between each factor and a reference sequence.

Standardize the data using Equation (1) to eliminate dimensional effects, then calculate the gray relational coefficients between each factor and reference sequence $x_{0}$ with Equation (2), and finally obtain the gray relational degree by weighted averaging of coefficients at each time point using Equation (3).

(4) $${x^{\prime }}_{i}(k)=\frac{x_{i}(k)-\min(x_{i})}{\max(x_{i})-\min(x_{i})}$$

(5) $$\xi_{i}(k)=\frac{\underset{i}{\min}\underset{k}{\min}\left|x_{0}(k)-{x^{\prime }}_{i}(k)\right|+\rho \underset{i}{\max}\underset{k}{\max}\left|x_{0}(k)-{x^{\prime }}_{i}(k)\right|}{\left|x_{0}(k)-{x^{\prime }}_{i}\left(k\right)\right|+\rho \underset{i}{\max}\underset{k}{\max}\left|x_{0}(k)-{x^{\prime }}_{i}(k)\right|}$$

(6) $$\gamma_{i}=\frac{1}{n}\sum_{k=1}^{n}\xi_{i}(k)$$

Gray correlation analysis compares relationships between pore parameters and compressive strength after freeze–thaw cycles. The results show that pore structure complexity and order significantly impact material strength (Table 5). Although pore morphology differs between Groups B1 and B4, Group B4 demonstrates a higher correlation of pore parameters under freeze–thaw conditions. This indicates greater pore structure stability in Group B4, enhancing the material’s compressive strength.

The proportion of large pores (B1: 0.803; B4: 0.817) and extremely low-abundance pore rates (B1: 0.822; B4: 0.837) correlate most strongly with compressive strength. These findings demonstrate synergistic enhancement of the structural load-bearing capacity by both pore characteristics. The high correlation of large pore proportion matches the literature findings [52], indicating that large pores increase the dominance of freeze–thaw damage.

The high correlation of extremely low abundance pore rates (average 0.829) confirms that dense, homogeneous material regions are key for mechanical stability. The morphology of these regions significantly enhances structural stability by restricting crack paths. Micropore proportion (B1: 0.785; B4: 0.803) and fracture dimension (B1: 0.763; B4: 0.778) show the next strongest correlations with compressive strength. These correlations indicate that pore size refinement and pore network complexity enhancement effectively strengthen the material.

## 4. Discussion

### 4.1. Freeze–Thaw Damage Mechanism Analysis

The strength degradation of cement–silt-modified eolian sand during freeze–thaw cycles involves complex physicochemical processes. These processes include internal structural changes, dynamic pore water variations, and cumulative thermal stress effects. This section discusses the freeze–thaw damage mechanisms in modified eolian sand from multiple perspectives.

#### 4.1.1. Physical Structural Changes

**Figure 16:**
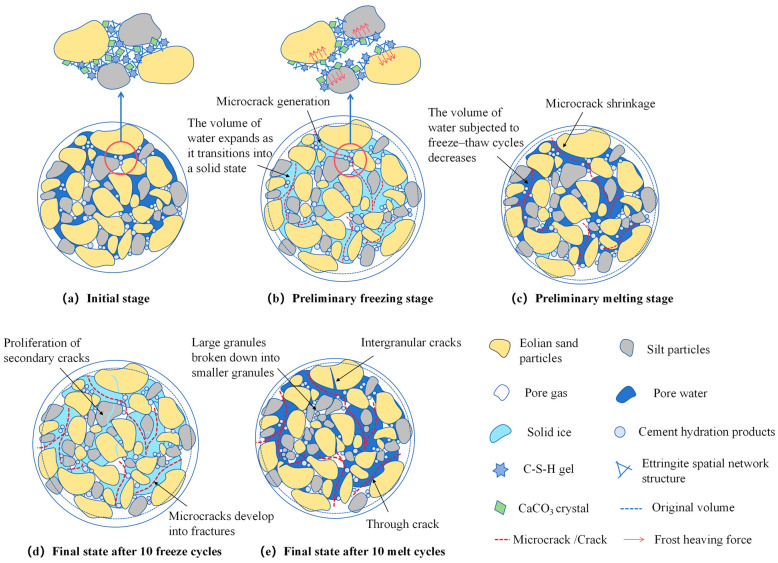
Schematic diagram of microstructural changes under freeze–thaw cycles.

Frost heave and thaw shrinkage cycles fundamentally alter material structures [53,54,55]. Below 0 °C, the pore water in modified eolian sand freezes. The water volume expands significantly during freezing, generating substantial frost heave stress [56,57,58]. This expansion enlarges small pores into larger ones, creating stress concentration around the expanded pores. When temperatures rise above freezing, the ice melts, causing specimen shrinkage. During this phase transition, the frost-heave-enlarged pores partially contract. However, non-uniform shrinkage induces pore wall and interfacial stress concentration [59]. Non-uniform shrinkage primarily stems from two aspects: distinct thermal expansion coefficients among modified eolian sand components (especially between cement and silt particles) and inherent variations in porosity and particle size distribution. Figure 16 illustrates these microstructural changes.

Stress concentration at pore walls damages the surrounding material and accumulates cyclic stress during the freeze–thaw. Pore structure loosening destabilizes the material’s interior, propagating microcracks along the pores. This increases the pore network complexity and irregularity, intensifying the stress concentration. Consequently, cracks develop progressively along the pore pathways, further weakening the overall structure.

#### 4.1.2. Chemical Change

Degradation of cement hydration products significantly contributes to freeze–thaw damage. Cement hydration reactions produce key compounds including calcium hydroxide (Ca(OH)_2_), calcium silicate hydrate (C-S-H), and ettringite. These products form the primary binding matrix and provide structural strength. However, freeze–thaw cycles severely challenge the stability of these hydration products. Some components, like ettringite, have a loose structure and high water content. This makes them vulnerable to temperature changes. Ice crystal growth in pores causes volume expansion. This expansion exerts strong physical forces on the hydrants. Consequently, their crystal structure may loosen or even disintegrate [25,60,61]. The disintegration of hydration products forms new micropores and cracks. These newly generated pores provide more space for water infiltration [62]. This exposure keeps the hydration products in a harsh environment of alternating wetting and freeze–thaw cycles, thereby accelerating their degradation. Additionally, these pores create conditions for subsequent ice crystal growth and volume expansion.

The impact of chemical erosion further accelerates the material’s degradation. In practical engineering environments, cement–silt-modified eolian sand is often used in areas with complex groundwater or surface water. These waters may contain soluble salts, such as sulfates and chlorides. During freeze–thaw cycles, ice crystal formation pushes dissolved salts into the unfrozen water film. This creates locally high-concentration salt solutions [18,19,24,63]. As temperatures rise, these solutions penetrate the material after the ice melts. They then react with the cement hydration products. These reactions lead to chemical erosion phenomena, such as sulfate attack and alkali–aggregate reactions. For example, sulfate reacts with Ca(OH)_2_ to form new ettringite crystals (Equations (4) and (5)). These new crystals have a much larger volume than that of the original ones. This generates greater internal expansion stress, triggering secondary damage and creating new microcracks [64,65]. Additionally, the chemical erosion process damages the C-S-H bonding system (Equation (6)), weakening the bonding strength between particles. Consequently, the overall strength and durability are reduced.

3CaO · Al_2_O_3_ · 6H_2_O + 3CaSO_4_ · 2H_2_O + 19H_2_O → 3CaO · Al_2_O_3_ · 3CaSO_4_ · 31H_2_O (7)

Ca(OH)_2_ + Na_2_SO_4_ · 10H_2_O → CaSO_4_ · 2H_2_O + 2NaOH + 8H_2_O (8)

Ca_3_Si_2_O_7_ · 3H_2_O + 6H^+^ → 3Ca^2+^ + 2SiO_2_ · nH_2_O + 3H_2_O (9)

### 4.2. Limitation

This study only explored the freeze–thaw resistance of a specific soil-to-sand ratio of 3:7. To more comprehensively reveal how the soil-to-sand ratio affects material freeze–thaw stability, future research will systematically investigate other key ratios, such as 2:8 and 4:6. This will improve our understanding of how different ratios impact freeze–thaw performance. Consequently, it will provide a more scientific basis for optimizing material ratio design.

## 5. Conclusions

We analyzed the effects of freeze–thaw cycles on the macro- and micro-properties of cement–silt-modified eolian sand using unconfined compressive strength tests, freeze–thaw cycling, and an SEM. This study also explored the freeze–thaw damage mechanism and drew the following conclusions:

(1) The compressive strength of cement–silt-modified eolian sand decreases gradually with increasing freeze–thaw cycles. Compared to the 5% cement mixture, the 8% cement mixture shows better freeze–thaw resistance. After 10 cycles, its compressive strength decreases by only 25%.

(2) As freeze–thaw cycles increase, the pore count in cement–silt-modified eolian sand rises continuously. Pore shapes deteriorate from circular/elliptical to elongated forms, and pore orientation shifts from ordered to random. These changes indicate microstructural damage caused by freeze–thaw cycles. This damage creates a more porous network structure, ultimately degrading mechanical properties.

(3) The 8% cement group shows stronger correlations between pore parameters during freeze–thaw cycles. The correlations among the large pore proportion, ultra-low abundance pore proportion, and compressive strength are particularly significant. This cement content mitigates the microcrack expansion and porosity increase caused by freeze–thaw cycles. Consequently, it delays material strength reduction.

## Figures and Tables

**Table 1:** Mechanical parameters of eolian sand and silt.

Type	Particle Density (g/cm^−3^)	Optimal Dry Density (g/cm^−3^)	Optimal Moisture Content (%)	Plastic Limit (%)	Liquid Limit (%)	Plasticity Index
Eolian sand	2.65	1.71	6.17	\	14.8	6.4
Silt	2.67	1.8	18	12.3	22.6	9.3

**Table 2:** Chemical composition and content of cement.

Composition	CaO	SiO_2_	Al_2_O_3_	Fe_2_O_3_	MgO	Na_2_O	K_2_O	SO_3_	Cl^−^	Free-CaO	Others	H
Content/%	57.57	19.5	6.45	3.08	1.21	0.25	1.35	2.01	0.03	0.81	0.77	6.97

**Table 3:** Test plan for UCS of cement-modified eolian sand.

Number	Soil–Sand Ratios	Cement Content/%	Curing Age/d
A1, A2, A3, A4, A5	2:8	5, 6, 7, 8, 9	7
B1, B2, B3, B4, B5	3:7		
C1, C2, C3, C4, C5	4:6		
B1, B4	Pure Eolian Sand	5, 8	

**Table 4:** Test program for unconfined compression of cement-modified eolian sand.

Number	Soil–Sand Ratios	Cement Content/%	Curing Age/d	Freeze–Thaw Cycle Times/Times
B1	3:7	5	7	0, 1, 2, 4, 7, 10
B4		8		
B1	Pure Eolian Sand	5		
B4		8		

**Table 5:** The correlation between aperture parameters and strength.

Aperture Parameters	Microporosity Percentage	Small-Hole Ratio	Medium Pore Percentage	Large Pore Percentage	Ultra-Low Abundance	Lower Abundance	Medium Abundance	Higher Abundance	Extremely High Abundance	Directed Probability Entropy	Fractal Dimension
5%	0.785	0.764	0.705	0.803	0.822	0.728	0.651	0.738	0.708	0.751	0.763
8%	0.803	0.769	0.672	0.817	0.837	0.733	0.672	0.762	0.743	0.702	0.778

## Data Availability

The original contributions presented in this study are included in the article. Further inquiries can be directed to the corresponding author(s).
